# Supplementary material for: Svep1 is a binding ligand of Tie1 and affects specific aspects of facial lymphatic development in a Vegfc-independent manner
Source: eLife. 2023 Apr 25;12:e82969. doi: 10.7554/eLife.82969 (PMC10129328; doi:10.7554/eLife.82969)
Supplement: Figure 8—source data 1. [file elife-82969-fig8-data1.pdf]

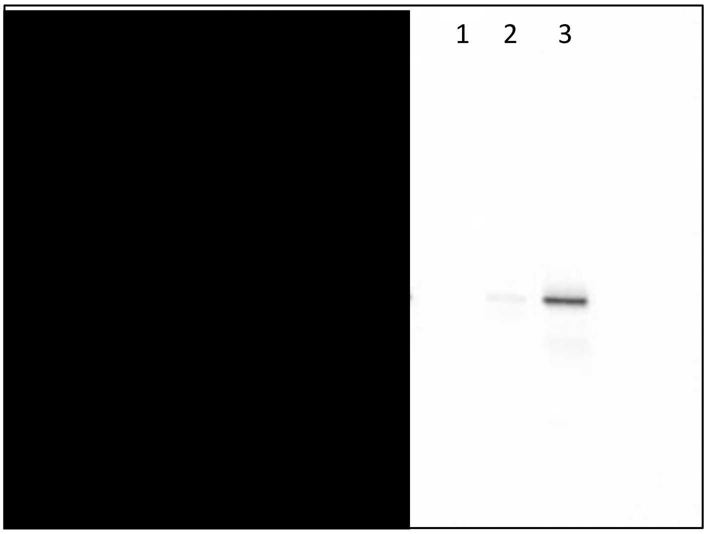

zfTie1 IP anti-HA 1sec Tie1 showed a strong band after pull down with Svep1 (3), but a faint band was also detectable in the control sample without Svep1 (2). This background band can be explained either by the immunoglobulin like domain of Tie1 binding to the Sepharose beads, or by inherent stickiness of Tie1 protein.

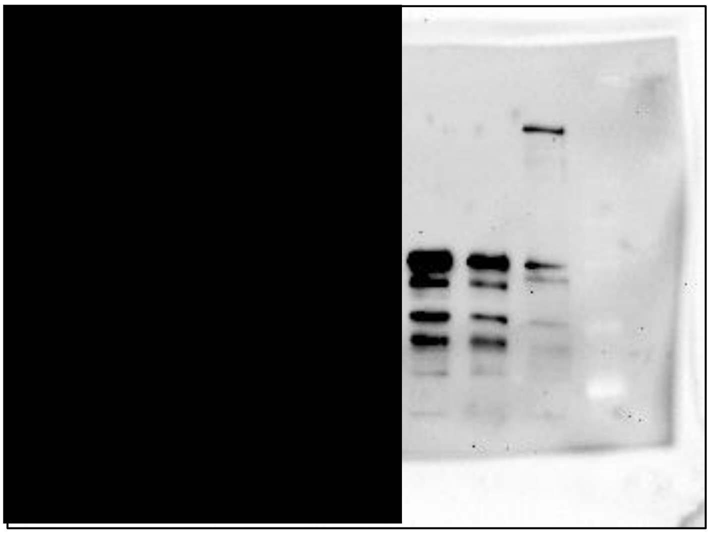

zfTie1 IP anti-HIS 233.6 sec

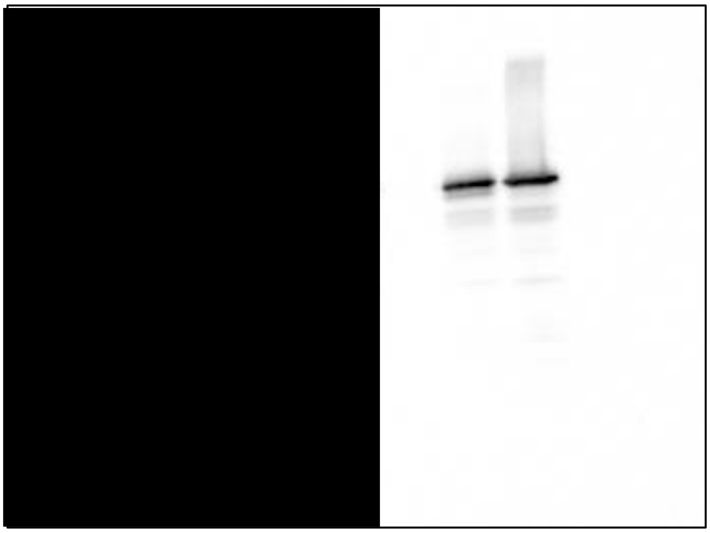

zfTie1 lysate anti-HA 34.2 sec

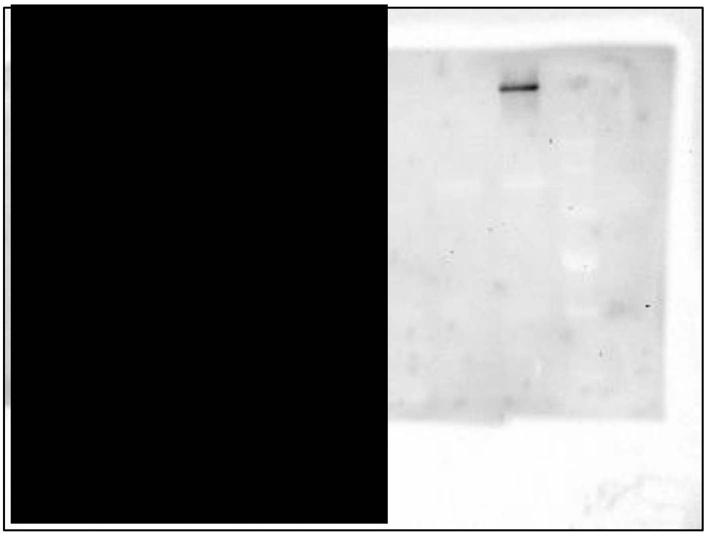

zfTie1 lysate anti-HIS 300 sec

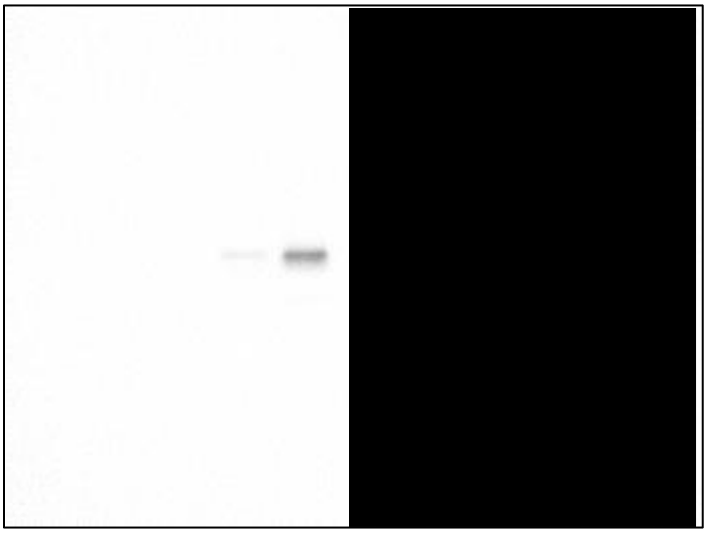

TIE1 Co-Immunoprecipitation anti-HA 7.1 sec

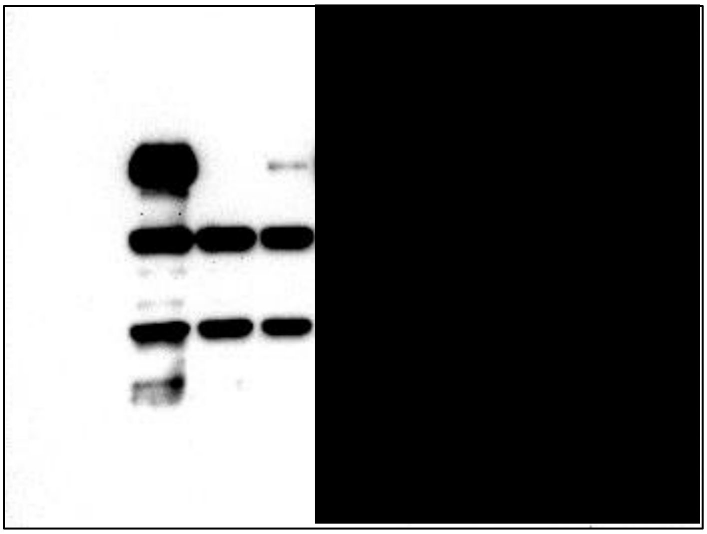

TIE1 Co-Immunoprecipitation Streptactin 338.9 sec

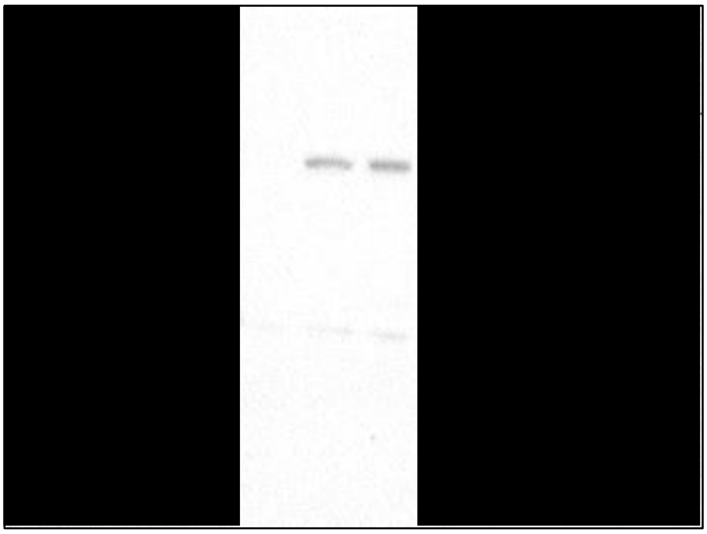

TIE1 Co-Immunoprecipitation lysate anti-HA 177.3 sec

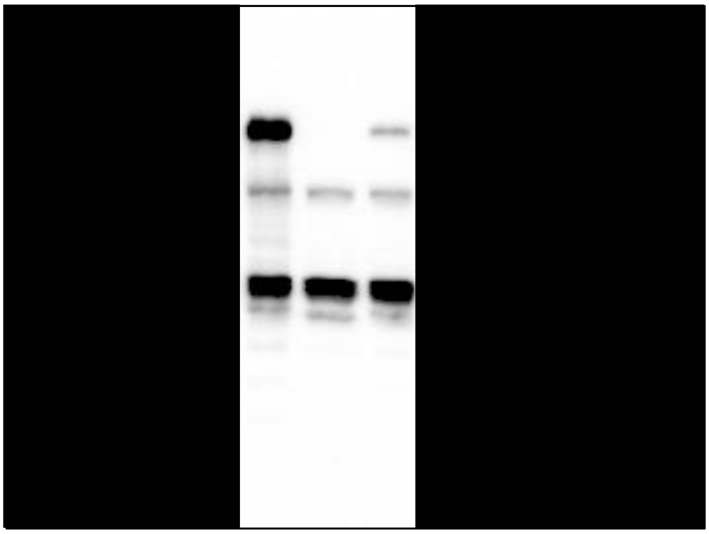

TIE1 Co-Immunoprecipitation lysate Streptactin 31.7 sec

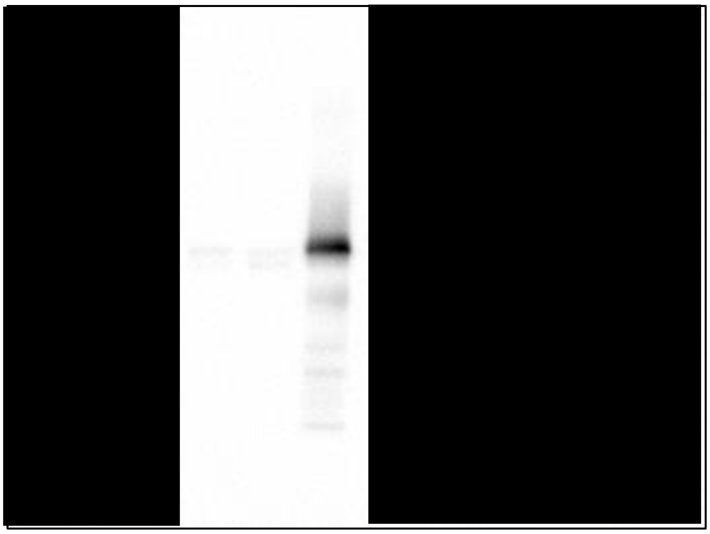

TIE1 Protein IP anti-HA 2.4sec

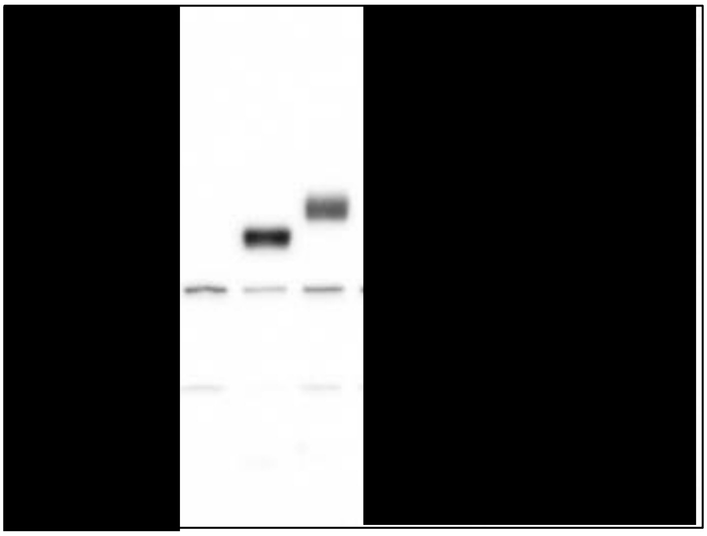

TIE1 Protein IP Streptactin 6.1 sec

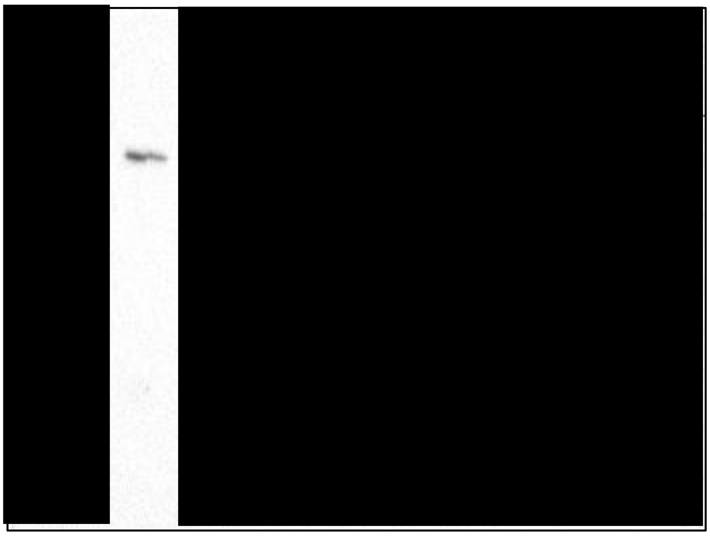

TIE1 Protein IP Lysate anti-HA 169.7 sec
