# Supplementary material for: Svep1 is a binding ligand of Tie1 and affects specific aspects of facial lymphatic development in a Vegfc-independent manner
Source: eLife. 2023 Apr 25;12:e82969. doi: 10.7554/eLife.82969 (PMC10129328; doi:10.7554/eLife.82969)
Supplement: Figure 8—figure supplement 1—source data 1. [file elife-82969-fig8-figsupp1-data1.pdf]

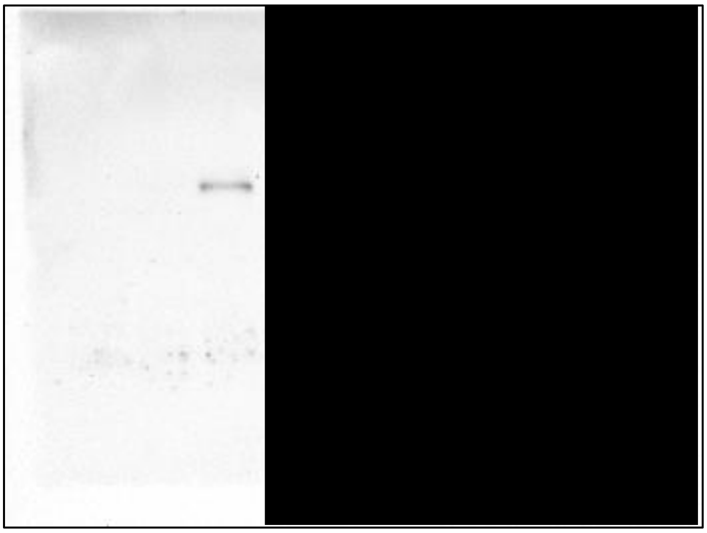

zfTie2 IP anti-HA 124.7 sec

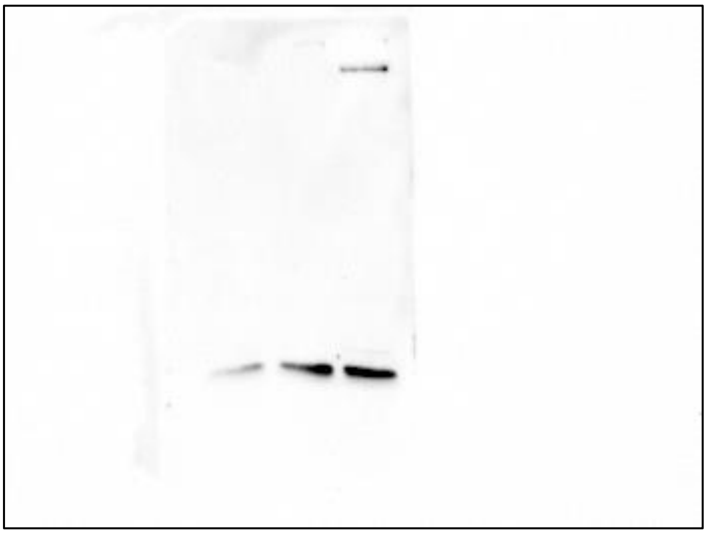

zfTie2 IP anti-HIS 133.9 sec

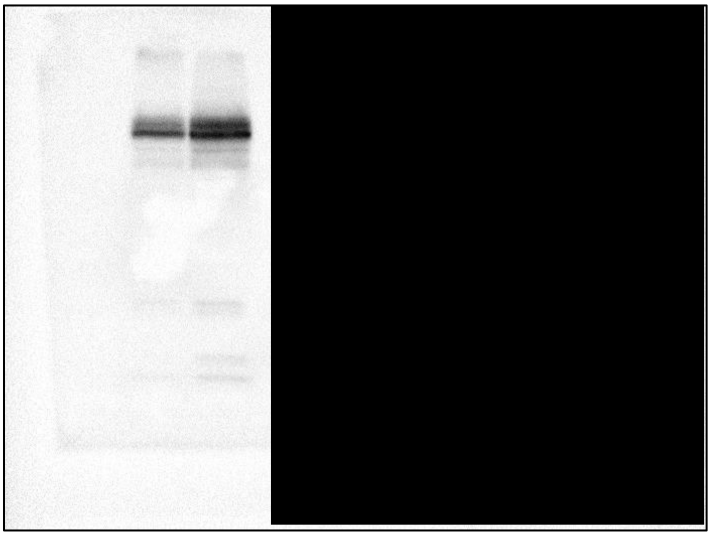

zfTie2 lysate anti-HA 41.4 sec

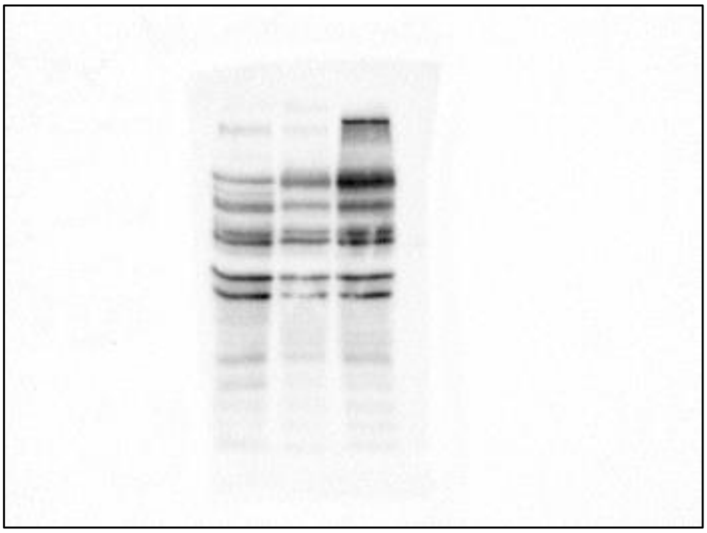

zfTie2 lysate anti-HIS 13.9 sec

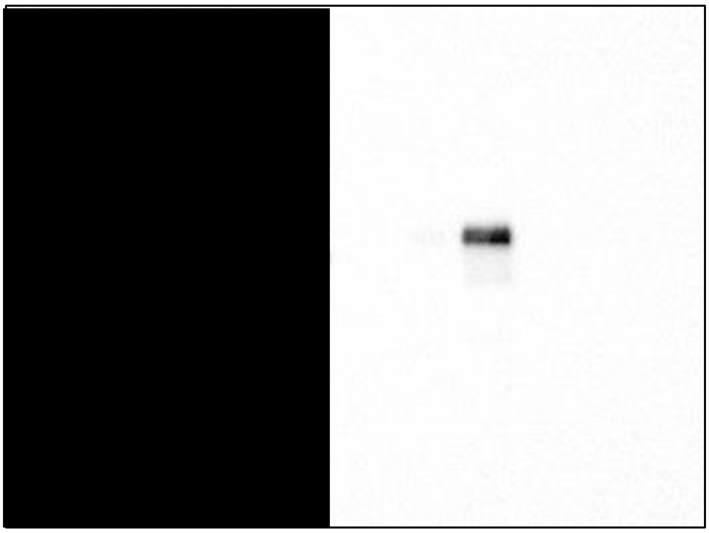

TIE2 Co-Immunoprecipitation anti-HA 4.1 sec

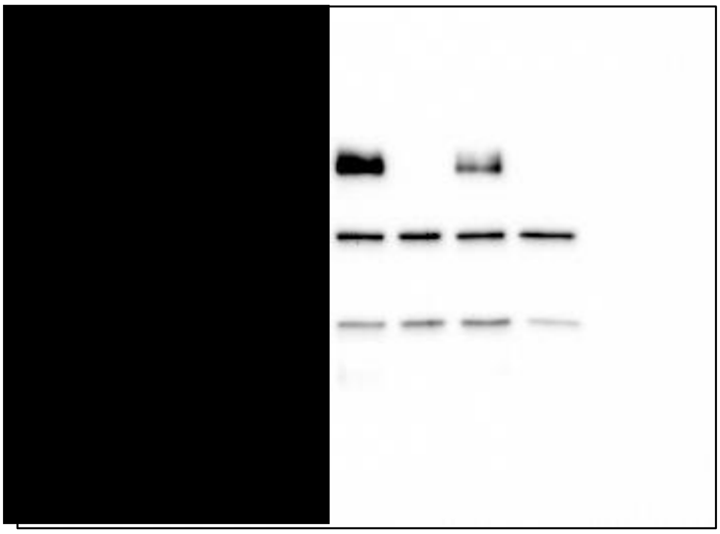

TIE2 Co-Immunoprecipitation Streptactin 47.1 sec

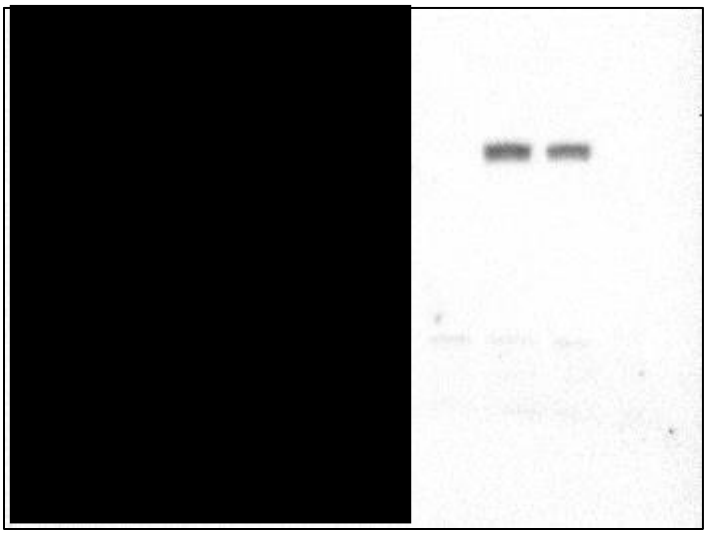

TIE2 Co-Immunoprecipitation lysate anti-HA 177.3 sec

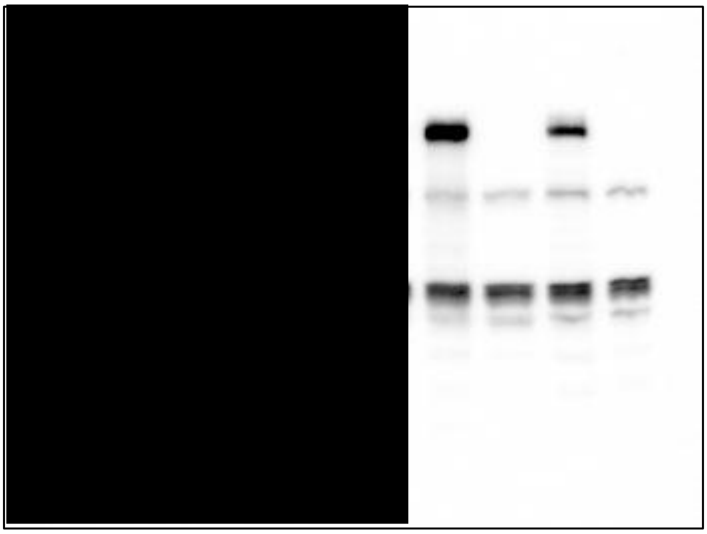

TIE2 Co-Immunoprecipitation lysate Streptactin 16.3 sec

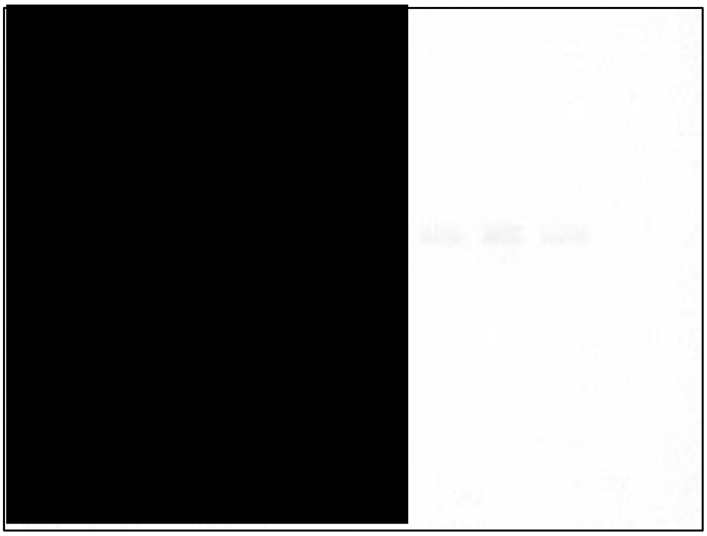

TIE2 Protein IP anti-HA 2.4sec

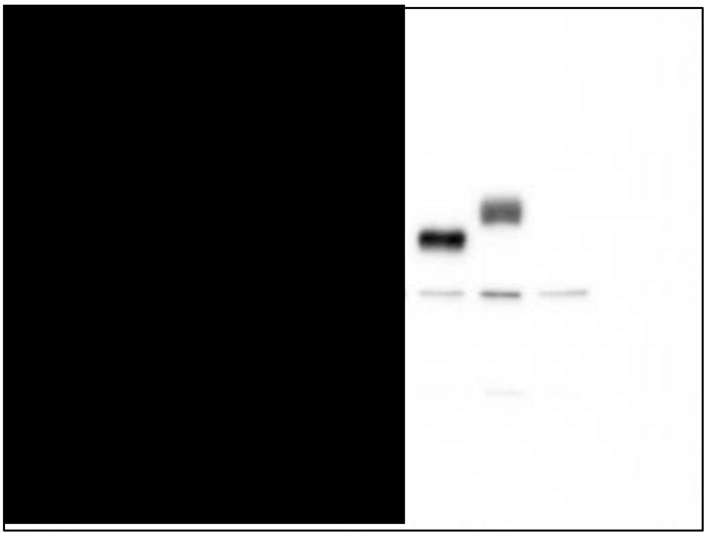

TIE2 Protein IP Streptactin 6.1 sec

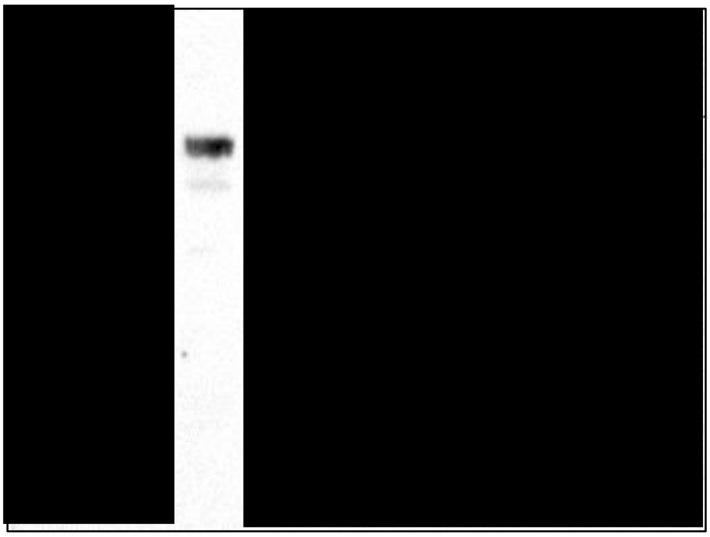

TIE2 Protein IP Lysate anti-HA 169.7 sec
